# Research on the impact of population mobility on green total factor productivity: A perspective from sustainable development

**DOI:** 10.1371/journal.pone.0337836

**Published:** 2026-01-02

**Authors:** Zuogong Wang, Xueli Yang, Linhai Wang, Zhuangfei Han

**Affiliations:** 1 Western China Green Development Strategy Research Institute, Guizhou Ufniversity of Finance and Economics, Guiyang, China; 2 Guiyang Jiao Tong Institute of Shared Finance, Guiyang, China; 3 School of Big Data Application and Economics, Guizhou University of Finance and Economics, Guiyang, China; 4 School of Economics, Guizhou University of Finance and Economics, Guiyang, China; 5 Department of Mathematics, University of Illinois Urbana-Champaign, Champaign, Illinois, United States of America; 6 Henan University School of Economics, Henan University, Kaifeng, China; Leshan Normal University, CHINA

## Abstract

The optimal allocation of the labor force through population mobility is a critical determinant of the synergistic economy, society, and environment, with substantial implications for Green Total Factor Productivity (GTFP). This study employs panel data from 2011 to 2020 encompassing 30 Chinese provinces, to measure the GTFP using the SBM-DDF model. We then investigated the network association characteristics of GTFP across provinces through social network analysis. Furthermore, fixed- and moderating-effects models are applied to empirically examine the impact of population mobility on GTFP, as well as the moderating roles of technological progress, financial development, and digital inclusive finance. These results indicate that China’s GTFP exhibits spatial clustering and regional disparities, with western and eastern regions being the primary sources and destinations of green resource flows, respectively. Population mobility enhances national GTFP, a finding that remains robust across several endogeneity and robustness tests. However, this effect is heterogeneous; it is significant in destination regions, eastern and western regions, and the post-industrialization stage, but negligible elsewhere. Furthermore, the moderating effect analysis shows that technological progress amplifies this relationship both nationally and in destination areas. Additionally, financial development strengthens the effect in destination regions, whereas digital inclusive finance strengthens the effect in origin regions. Recognizing population mobility as a globally pervasive phenomenon, this study highlights its functional role and advances our understanding of its relationship with sustainable development.

## 1 Introduction

Since the beginning of the 21st century, resource depletion and environmental degradation have posed significant global challenges that require consideration. Consequently, ensuring population security and pursuing sustainable development in both the economy and the environment have become crucial concerns on a global scale [1]. In response, the *United Nations’ 2030 Agenda for Sustainable Development* (2015) established 17 sustainable development goals (SDGs) aimed at promoting the harmonious development of the population, economy, environment, and society. Sustainable development seeks to ensure intergenerational equity by maintaining a dynamic equilibrium between the economic, social, and environmental systems within a given region. However, persistent ecological degradation, resource overexploitation, and climate change threaten this equilibrium [2]. Harmonizing economic growth and ecological sustainability to ensure coordinated development is a critical challenge for nations worldwide. The key to achieving sustainable development that harmonizes economic growth with environmental protection is enhancing the Green Total Factor Productivity (GTFP) [3]. Unlike conventional productivity measures, the GTFP incorporates energy consumption and environmental pollution as undesired outputs while evaluating how efficient inputs such as capital, labor, and technology generate the desired outputs [4–6]. China, the world’s second largest economy and a nation with substantial population mobility, traditionally relies on the consumption of natural and human resources as drivers of economic growth. The presence of ample and relatively inexpensive labor has propelled the advancement of the manufacturing sector. Capitalizing on its natural resource endowments and demographic dividends, China has embraced a production model that prioritizes intensive labor and resource inputs to attain high output. However, this approach poses obstacles in the pursuit of sustainable development.

According to *the 2020 Seventh National Population Census of China*, the mobile population reached 376 million, representing 26% of the total population, a 69.73% increase from 2010. Population mobility facilitates the movement of capital, technology, and other production factors, thereby fostering adjustments to economic and industrial structures. This plays a crucial role in improving factor productivity and alleviating environmental pressure. However, its impact on GTFP is multifaceted. On the one hand, mobility breaks down economic and cultural barriers to exchange, reduces transaction and temporal costs, and correlates with lower energy consumption, PM2.5, NOx emissions, and carbon footprints [7]. Furthermore, it elevates investment efficiency through technology diffusion, knowledge spillovers, and material capital accumulation [8]; accelerates digital economy growth and regional innovation capacity [9]; and strengthens urban economic resilience [10], collectively underpinning GTFP improvement. On the other hand, research also suggests that population mobility’s economic benefits often rely on energy-intensive industries and infrastructure [8], exacerbating air pollution and environmental degradation [11]. Moreover, mobility reduces kin and reciprocal altruism, encourages self-interest in collective welfare, and diminishes pro-environmental behavior, thereby intensifying ecological strain [12]. Additionally, their impact on carbon emissions depends on the skill structure of migrants [13]. Thus, the effect of population mobility on GTFP remains uncertain. Given the fading demographic dividend, persistent large-scale population mobility, and sustainability imperatives, studying the impact of population mobility on GTFP is critically important.

Research on population mobility and GTFP reveals four key strands. The first examines spatial evolution of GTFP. Researchers have utilized kernel density functions and Markov chains to analyze the spatial evolution of GTFP [14]. However, there is a dearth of studies that utilize social network models to examine interregional GTFP positioning and its dynamics, leaving a gap in the understanding of relational spatial shifts. Second, research on the economic impact of population mobility predominantly highlights its positive contributions to growth [9,15], labor market flexibility [16], regional development [17], and resource allocation efficiency [18]. Third, the nexus between population mobility, productivity, and environmental sustainability reveals divergent perspectives. Most studies have emphasized these benefits. For instance, mobility-induced agglomeration reduces operational costs, energy consumption, and carbon emissions [19,20], because migrants tend to exhibit stronger environmental consciousness and adopt energy-saving behaviors [21]. Additionally, the spatial mobility of populations not only fosters cultural diversity, thereby laying the foundation for sustainable development [22], but also strengthens intercity collaboration and knowledge spillovers, thereby enhancing urban productivity [23]. However, dissenting views argue that mobility-driven population imbalances and the resulting diseconomies of scale may hinder productivity [24,25], particularly because their economic benefits are often derived from energy-intensive industries that exacerbate environmental degradation [8]. Some studies have suggested that the environmental effects of mobility vary according to the demographic characteristics. Destination areas often increase energy consumption and emissions of pollutants, such as SO_2_ and NO_X_, particularly when populations flock to destinations, resulting in an environmental burden [26]. Only high-skilled population mobility increases carbon emissions, while ordinary-skilled population mobility reduces them [13]. Fourth, investigations into GTFP determinants span technological progress [27], financial development [28], energy consumption structure [29], human capital [30], and economic policies [31]. However, in the short term, relying on adjustments in the energy structure to improve GTFP can be quite challenging. As such, external factors such as technological progress should be considered important [27].

Although existing research has yielded substantial insights, several gaps remain that warrant further investigation. First, although existing studies have employed various modeling approaches to analyze spatial evolution of GTFP, limited attention has been paid to interregional spatial correlations and dynamic positional changes. Second, while the literature provides substantial evidence regarding both the economic and sustainable development effects of population mobility, as well as the determinants of GTFP, scholarly consensus remains elusive. Most studies examined these dimensions in isolation, with limited research addressing their combined effects on GTFP coordination. Notably, the role of population mobility as a determinant of GTFP has received inadequate systematic analysis. Third, the relationship between labor, technology, capital, and economic growth has long been central to economic growth theory, and the potential synergistic effects of population mobility, technological progress, and financial capital in enhancing GTFP remain underexplored. Therefore, this study addresses three critical research questions: (1) What spatial correlation patterns characterize China’s provincial GTFP? (2) Does population mobility influence GTFP growth positively? (3) Do technology, capital, and labor exhibit synergistic effects on GTFP enhancement? Methodologically, we employ social network analysis (SNA) to examine interprovincial GTFP network correlations, followed by two-way fixed- and moderating-effects models to analyze population mobility’s impact and its interaction with technological progress and financial capital. Our findings reveal that provincial GTFP demonstrates significant spatial interdependence with distinct agglomeration effects among neighboring provinces and uneven regional development. Population mobility exhibited an overall positive effect on GTFP, although this impact was heterogeneous – significant in destination regions, the eastern and western areas, and the post-industrialization stage. Technological progress exerts a positive moderating effect on the relationship between mobility and GTFP in both national and destination regions. In contrast, financial development exhibits a positive moderating effect solely in destination regions, while digital inclusive finance plays a comparable role in origin regions.

This study contributes to the literature in three ways. First, it employs the SBM-DDF-GML measurement of GTFP and applies SNA to explore the social network structure characteristics and spatial evolution trends of GTFP. This approach reveals the relative positioning of GTFP across regions, its dynamic changes over time, and the interactions among regions, thereby providing a clearer depiction of regional GTFP trends and their interdependencies. Second, this study integrates the economic and environmental effects of population mobility to elucidate its relationship with GTFP. While existing literature has examined the impact of population mobility on either environmental quality or productivity in isolation, few studies have simultaneously considered both dimensions, particularly their combined effects on GTFP. Moreover, previous research has predominantly focused on contexts such as the international community [32], the UK [33], Palestine [20], Ethiopia [34], and Africa [35]. Given China’s unprecedented scale of population mobility and its potential economic and environmental implications, this study innovatively incorporates population mobility into GTFP to integrate the economic and environmental benefits associated with population mobility. This approach not only advances theoretical frameworks related to demographic structure and sustainable development but also expands the green dimension of economic growth theory. Moreover, it provides a novel perspective for analyzing the “population-environment” nexus within the economic growth theory and enriching the application of the “demographic dividend” concept in the realm of sustainability. Third, grounded in endogenous growth theory, which internalizes technological progress and human capital as drivers of economic growth, this study systematically examines the synergistic effects of population mobility, technological progress, financial development, and digital inclusive finance on GTFP enhancement. Using the moderating effects model, we assessed the moderating roles of these factors in shaping GTFP dynamics. This approach provides a comprehensive analytical framework and multidimensional policy insights to understand the dynamics of sustainable development.

The remainder of this paper is organized as follows. Section 2 presents a literature review and research hypotheses. Section 3 describes the research methodology and variables. Section 4 presents the empirical results. Section 5 discusses our findings and their implications. Section 6 provides the research conclusions and suggestions.

## 2 Literature review and theoretical analysis

GTFP is widely recognized as a pivotal indicator for reconciling the tension between economic growth with environmental sustainability [4]. Many post-industrialized economies increasingly face challenges such as declining birth rates and the erosion of traditional demographic dividends. Consequently, leveraging population mobility is increasingly regarded as a strategic imperative for enhancing GTFP [8,35]. The New Economic Geography emphasizes the economies of scale generated by the spatial agglomeration of population and industries, postulating population mobility as a critical pathway for alleviating environmental pollution and enhancing energy efficiency [36]. Complementing this, Human Capital Externalities suggests that such mobility fosters innovation effects through knowledge spillovers, which facilitate the transfer of tacit knowledge and the diffusion of green technologies [13]. Furthermore, Endogenous Growth theory considers the accumulation of human capital—a process significantly accelerated by population mobility—to be a fundamental driver of technological progress and regional innovation [37]. Collectively, these theories suggest that population mobility can simultaneously stimulate economic growth and facilitate sustainable development.

### 2.1 Spatial evolution of GTFP

Owing to the spatial interaction of policies and factors, an analysis of GTFP cannot be conducted independently. Rather, it must account for the interactions that arise from its own GTFP and those of other regions under the network relationships formed by regional linkages. Previous studies found an increasing spatial agglomeration effect in GTFP [38]. However, conventional regression analyses primarily examine individual study subjects in isolation, neglecting the evolving dynamics of GTFP development across all interconnected regions. Furthermore, the nonparametric kernel density function [39] was employed to analyze the dynamic evolution of regional disparities in GTFP over time and their spatial convergence. Nevertheless, these methods provide a limited exploration of the scale and structural characteristics of GTFP networks. In contrast, SNA can be used to analyze the gridded evolution process of the spatial structure and regional interactions of GTFP over a given period or different time dimensions. This approach fully demonstrates the position, transmission direction, and interaction of GTFP within all regions. For instance, Chen et al. (2022) employed SNA to investigate the general situation of agricultural GTFP in China and the status of each research object in the network [40]. Similarly, He and Li (2024) constructed population mobility network indicators using SNA, demonstrating how mobility networks enhance GTFP by facilitating technology diffusion, knowledge exchange, and material capital accumulation through improved investment efficiency [8].

### 2.2 Population mobility and GTFP

Population mobility, which encompasses both permanent and temporary migration, has been studied extensively, particularly regarding its marginal effects on productivity and sustainability. In this context, this study extends the existing research by examining the impact of temporary migration on GTFP. Specifically, it explores the relationship between population mobility and GTFP from two perspectives: population mobility and productivity, and population mobility and environmental sustainability.

Population mobility influences productivity through various mechanisms including agglomeration effects and the externalities of knowledge and technology. Academic consensus has confirmed that population mobility contributes to productivity improvements. Lewis (1954) [41] and Ranis and Fei (1961) [42] pioneered the concept that surplus rural labor flows to cities, leading to capital accumulation and economic growth. Population mobility facilitates the agglomeration of talent, capital, and industries, thereby enhancing regional human capital, stimulating innovation, fostering specialized labor division patterns, and generating economies of scale through specialization [23]. Harris and Todaro (1970) considered the issue of unemployment in developing countries. They argue that migration plays a more significant role in these countries than in developed nations with more efficient labor markets [43]. By improving the matching of labor supply and demand in a rigid labor market, population mobility enhances the efficiency of labor resource allocation. Additionally, population mobility generates positive externalities through technology diffusion and environmental knowledge transfer. This improves investment efficiency through network technology diffusion effects, knowledge spillovers, and material capital accumulation [8]. This process fuels the development of the digital economy and regional innovation capacity [9] and improves the economic resilience of the city [10], thereby providing support for GTFP. In contrast, large-scale population mobility can also lead to an uneven population distribution and give rise to a series of “urban disease” problems. Megacities become overcrowded, resulting in resource misallocation and inefficient scaling, which hinders economic growth [44]. Furthermore, there is some uncertainty regarding the relationship between population mobility and productivity owing to differences in sample periods, labor quality, and regional resource endowments. For instance, Ma and Tang (2020) suggested that population mobility between cities promotes local welfare improvement, and overall productivity mainly benefits from population inflow [45], while Gören (2014) proposed that population mobility does not significantly impact economic growth [25].

Regarding population mobility and environmental sustainability, numerous studies indicate that population mobility promotes population concentration, reduces operational and time costs, and improves energy efficiency, thereby reducing energy consumption and pollutant emissions [7,19,20]. This phenomenon may be explained by the tendency of mobile populations to exhibit heightened environmental awareness, prioritize sustainable development, and generate less waste and pollution [33]. Hoel and Shapiro (2001) argued that population mobility fosters a convergence of interests among regions, prompting each region to pursue policies that optimize aggregate welfare while advancing its individual developmental objectives. This alignment of interests mitigates transboundary environmental pollution and facilitates sustainable development [46]. Supporting this perspective, Rees et al. (2016) used population data from 91 countries worldwide and found that population mobility plays a critical role in redistributing populations, altering population concentration patterns, and creating favorable conditions for the transformation and upgrading of industries toward cleaner sectors dominated by services, knowledge, and culture [47]. However, some studies suggest that population growth and urbanization precipitated by population mobility increase the pressure on the urban carrying capacity, thereby exacerbating energy consumption and carbon emissions [8,26] and inducing short-term labor intensification in the manufacturing sectors which poses challenges to sustainability [48]. Based on the above analysis and considering that large-scale population mobility in China can lead to improved resource utilization efficiency and the potential for agglomeration effects and externalities, we propose Hypothesis 1.

**Hypothesis 1**. *Population mobility promotes the enhancement of GTFP, with a more pronounced effect observed in destination compared to origin regions*.

### 2.3 Mechanism analysis of population mobility on GTFP from the perspective of sustainable development

Sustainability of technological progress. Solow (1957) proposed the Solow growth model, which suggests that technological progress, as an exogenous factor, is a core driver of economic growth [49]. Technological progress is associated with improvements in green efficiency, reductions in carbon emissions, knowledge spillover, and broader contributions to sustainable development [50]. It has a positive marginal impact on GTFP and serves as a source of momentum for its improvement. Population mobility and technological progress interact synergistically as two fundamental production factors that enhance GTFP. Population mobility facilitates the exchange and dissemination of knowledge, thereby contributing to technological progress, particularly high-quality technological progress [51,52]. Technological progress offers opportunities for mobile populations to continuously expand their knowledge and capabilities, facilitates the interaction and integration of various innovative elements, and generates extensive knowledge spillover effects [53]. The integration of emerging technologies with traditional industries generates novel technological applications and business models, thereby promoting their transformation and upgradation. Technological convergence promotes production intensification through advanced technologies, simultaneously enhancing green efficiency and reducing both aggregate energy consumption and energy intensity levels. These synergistic effects support progress toward sustainable development objectives and improvements in total factor productivity [54]. However, the effectiveness of technological innovation depends on appropriate resource endowments and the level of economic development. Studies find an ambiguous relationship between technological innovation and GTFP [55]. Urban technological innovation may either promote [6,29] or inhibit GTFP improvement [56]. Considering the reality of China’s large-scale population mobility and urbanization, as the scale of factor markets in destination areas expands and regional agglomeration levels improve significantly, the promotion of green efficiency, carbon reduction, and knowledge spillover effects is quite prominent. However, in some remote areas where there is a hollowing-out of the population, coupled with the high initial cost of green technology application and the existence of the digital divide, the exchange and dissemination of green technology may be hindered, resulting in an inability to improve GTFP [55]. Based on the above analysis, Hypothesis 2 is proposed.

**Hypothesis 2**. *The promotion effect of population mobility on GTFP is moderated by technological progress, with a more pronounced effect observed in destination compared to origin regions*.

To assess whether financial capital and population mobility synergistically enhance GTFP, this study adopts a dual-dimensional analysis of the financial system, incorporating both aggregate scale and structural composition. Specifically, financial development is employed as a measure of the scale of the financial system, whereas digital inclusive finance is used to quantify its structure of the financial system.

Regarding aggregate scale, financial development exhibits a “center-periphery” spatial distribution structure, which aligns with new economic geography’s location theory [57]. Central cities that attract population inflows develop financial agglomerations through favorable technological environments, economic scales, and other prerequisites. These agglomerations strengthen the exchange and imitation of advanced technologies between industries, reduce information asymmetry, optimize the efficiency of financial resource allocation, and generate technology spillovers [58]. Moreover, with the promotion of Environmental, Social, and Governance (ESG) and the development of green finance, banks have strengthened their review of corporate ESG investments, reduced information asymmetry, and alleviated corporate financial constraints. Therefore, Chinese companies are incentivized to adopt more ESG activities and replace outdated and intensive production technologies with green and clean ones [59]. However, the opposite view states that under limited financial resources, a serious mismatch in production factors inhibits the improvement of total factor productivity in developing countries [60]. Financial development stimulated by economic growth may also stimulate investment in heavy industries. This not only increases carbon emissions but also hinders the market exit of high-polluting enterprises. As a result, the improvement in GTFP is inhibited [8]. Therefore, in areas where population outflows occur, such as rural peripheral areas, inadequate financial development often leads to a lack of funding, distorted financial prices, and financing constraints for technology-based private enterprises. In addition, the outflow of highly-skilled personnel impedes green technology and efficiency improvements.

Structurally, digital inclusive finance has emerged as an innovative financial model driven by rapid digital technological advancement. There is a strong link between population mobility and the development of digital inclusive finance [61]. Digital transformation increases the demand for specific skilled labor, while mobility accelerates the application and popularization of digital technology, fostering digital economic growth and regional innovation [9]. This synergy enhances production efficiency, optimizes resource allocation, and upgrades industrial structures, ultimately boosting GTFP. Due to China’s special institutional environment, the areas of population outflow tend to be mostly rural. Digital inclusive finance integrates digital technology and inclusive finance, enhances capital market activity, and supports sustainable business development while addressing the cost-related limitations of traditional financial services in rural areas. It promotes the accumulation of human capital in rural areas [62,63], improves the mismatch between capital and labor [64], and facilitates environmental quality improvement [65]. Based on the above analysis, Hypothesis 3 is proposed.

**Hypothesis 3**. *The promotion effect of population mobility on GTFP is moderated by financial capital, with a significant moderating effect of financial development observed in destination regions, whereas digital inclusive finance exerts moderating effects in origin regions*.

Given that technological progress and financial development may vary in the impact of population mobility on GTFP across different developmental stages, it is necessary to further explore which factor is more dominant. The modified Solow model posits diminishing marginal returns on capital, while technological progress sustains per capita output growth as an exogenous variable. Technological progress is an intrinsic driver of economic growth [66]. Technological progress can be classified into indigenous and imitation innovation, with different choices at different stages of development; however, the advantage of latecomers is evident [67]. Notably, Bartelsman and Doms (2000) found that 75% of industry-level TFP growth comes from imitation innovation by incumbent firms, while only 25% comes from indigenous innovation by new entrants [68]. Evidence indicates that long-term sustained economic growth is more fundamentally attributable to productivity improvements propelled by technological progress than to capital accumulation alone [49]. Méon and Weill (2010) examined the relationship between financial development and technological efficiency in 45 countries and found that financial development improves technological efficiency and enhances GTFP only when the economy surpasses a certain level [69]. This effect is attributed to improvements in the financial system, a reduction in financial frictions, the mitigation of factor distortions, and the enhancement of capital allocation efficiency. Conversely, X Zhao et al. (2022) found that financial development inhibits GTFP improvement [70]. Based on this, Hypothesis 4 is proposed.

**Hypothesis 4**. *The synergistic effect of technological progress in the process of population mobility on GTFP enhancement gradually increases, while the role of financial development is weakened*.

## 3 Methods, models, and variables

### 3.1 Social network analysis

SNA employs graph theory techniques and algebraic models to define the relationships among units and investigate the opposite effects of connection models on the units [71]. SNA was built on the establishment of an economic connection model, specifically, a gravity matrix. The basic gravity model was set as shown in equation (1).


$$F_{ij}=k_{ij}\frac{M_{i}M_{j}}{D_{ij}^{b}}$$ (1)

$F_{ij}$ represents the gravitational strength between provinces i and province j. $M_{i}$ and $M_{j}$ are the respective masses of province i and province j, whereas $D_{ij}$ represents the spherical geographical distance between them. The parameter b is the distance attenuation coefficient, which is set to 2. $k_{ij}$ is an empirical constant that represents the gravitational coefficient between the two provinces, reflecting the contribution of province i to their bilateral connections. Given the causal relationship between the GTFP of provinces, the connections between any two provinces are inherently asymmetric. Equation (2) represents an extended gravity model.


$$y_{ij}=k_{ij}\frac{GTFP_{i}\times GTFP_{j}}{D_{ij}^{2}}\;k_{ij}=\frac{GTFP_{i}}{GTFP_{i}+GTFP_{j}}$$ (2)

Where $GTFP_{i}$ and $GTFP_{j}$ represent the GTFP of province i and j, respectively. Using an adjusted gravity model, we constructed a gravity matrix to analyze the development of the interprovincial GTFP. The average value of each row was taken as the threshold, where a gravity value of 1 was assigned to entries above the threshold, indicating the spatial spillover of GTFP development from the row province to the column province. Conversely, entries below the threshold were assigned a gravity value of 0, indicating the absence of a spatial spillover.

### 3.2 Model construction

To test Hypothesis 1 and explore the impact of population mobility on GTFP, we first construct a benchmark econometric model, as shown in equation (3).


$$\ln GTFP_{it}=\alpha_{0}+\alpha_{1}POPM_{it}+\theta_{i}X_{it}+\mu_{i}+\nu_{t}+\varepsilon_{it}$$ (3)

In this context, i represents provinces (autonomous regions, and municipalities directly under the central government), t denotes the year. The dependent variable $GTFP_{it}$ represents the level of development of GTFP. The main explanatory variable, $POPM_{it}$ represents population mobility, and is measured by the growth rate of the mobile population. $X_{it}$ encompasses a series of factors that may influence the GTFP. $\mu_{i}$ and $\nu_{t}$ represent province and year fixed effects, respectively, while $\varepsilon_{it}$ accounts for random disturbances.

We examine the mechanism of population mobility’s effect on GTFP, and test Hypotheses 2 and 3, by introducing moderating variables, namely technological progress (RD), financial development (FIN), and digital inclusive finance (DFIN), as well as an interaction term between population mobility and these variables in equation (3) to construct equation (4), which enables us to investigate whether a synergistic effect exists between technological progress, financial development, digital inclusive finance, and population mobility in promoting GTFP.


$$\ln GTFP_{it}=\alpha_{0}+\alpha_{1}POPM_{it}+\alpha_{2}M_{it}+\alpha_{3}POPM_{it}\times M_{it}+\theta_{4}X_{it}+\mu_{i}+\nu_{t}+\varepsilon_{it}$$ (4)

Where $M_{it}$ represents the moderating variable, encompassing technological progress, financial development, and digital inclusive finance. The term $POPM_{it}\times M_{it}$ indicates the moderating effect of the variable. The meanings of the remaining variables are identical to those in equation (3).

### 3.3 Variable selection and data description

Explained variable: Green total factor productivity (lnGTFP). Consistent with contemporary approaches [4–6], the measurement of GTFP should consider sustainable development in the economic, resource, and environmental dimensions. We selected 30 provinces in China (excluding Tibet, Hong Kong, Macao, and Taiwan) as our sample for 30 Decision Making Units. We chose the year-end number of employees in urban units, fixed asset investment, and total energy consumption as input indicators for the three factors of labor, capital, and natural resources. In terms of output indicators, we used two expected measures: GDP as the ideal output and actual GDP converted by constant prices as comparable across periods. In addition, we introduced the green areas of built-up areas as a characteristic variable for measuring social benefits. Furthermore, we incorporate three non-expected output indicators. Given the significant challenges posed by water and air pollution in China, we employed the quantities of industrial “three wastes” emissions, namely industrial wastewater discharge, industrial sulfur dioxide emissions, and industrial smoke (dust) emissions, as proxies for non-expected outputs. Based on the obtained input-output data, we used MATLAB and the SBM-DDF-GML model to calculate the GML index for each province from 2011 to 2020 [61]. The calculated GML index reflects only the rate of change in the GTFP. By setting the GTFP of each province in the base period to 1 and leveraging the cumulative nature of the GML index, we obtained the GTFP for each period [72].

Core explanatory variable: Population mobility (POPM). The relaxation of China’s household registration system has enhanced the accuracy of permanent resident statistics in capturing regional population mobility. As net population inflow provides a more precise reflection of interregional labor force movements, given that regions simultaneously experience inflows and outflows, we adopted the net population migration rate as the primary metric for assessing population mobility [9]. The net population migration rate is defined as the difference between the annual permanent population and the population growth due to natural increases. A positive value indicates a net population inflow, whereas a negative value indicates a net population outflow. For details, refer to equation (5), where all population types refer to the permanent population. Additionally, it should be noted that population mobility measurement is based on provincial-level administrative divisions and annual data. Therefore, short-distance population movements were not included, which may have led to an underestimation of the true scale of population mobility.


$$POPM_{it}=\frac{population_{i}-population_{j}-(births_{i}-deaths_{i})}{population_{j}}$$ (5)

Where $population_{i}$ and $population_{j}$ represent the numbers of permanent residents at the end and beginning of the year, respectively. $births_{i}$ and $deaths_{i}$ denote the number of births and deaths in that year.

Mechanism variables: Technological progress (RD). Research and development (R&D) investment is a fundamental indicator of technological innovation capabilities. The growth rate of internal expenditures on R&D funds by industrial enterprises above a certain scale is used as a proxy variable for technological progress [27]. Financial development (FIN). The financial industry’s value-added reflects the total value created by various financial institutions in providing financial services in a given year. This can be used to effectively measure financial development. Therefore, the ratio of the value-added of the financial industry to GDP is used to measure financial development [6], and its growth rate is calculated. Digital inclusive finance (DFIN). Digital inclusive finance is represented by the *Digital Financial Inclusion Index*. The index provides a standardized metric for assessing regional disparities in digital inclusive finance development across provinces [64].

Control variables. To minimize the estimation bias caused by omitted variables, the selection of control variables was guided by prior research [8,24,29]. Economic development was represented by the natural logarithm of per capita GDP at the regional level (lnPGDP). The extent of urban infrastructure construction was measured by per capita road mileage (INFRA). The level of urbanization was measured as the ratio of the urban population to the total population (URB). Financial support from local governments was represented by the natural logarithm of expenditure on scientific research funded by local finances (lnGOV). The level of industrialization was represented by the ratio of secondary industry to GDP (IND). Table 1 presents the key variables used in the empirical analysis.

**Table 1:** Summary of the empirical variables.

Category	Variable name	Symbol	Measurement	Source
Dependent variable	green total factor productivity	lnGTFP	SBM-DDF-GML model	China City Statistical Yearbook Statistical bulletins of each province; China Industrial Statistical Yearbook
Independent variable	population mobility	POPM	The net population migration rate	China City Statistical Yearbook
Mechanism variables	Technological progress	RD	Growth rate of internal R&D expenditures in large-scale industrial enterprises	China Science and Technology Statistical Yearbook
	Financial development	FIN	Value added of the financial industry/ GDP	China Financial Yearbook
	Digital inclusive finance	DFIN	Total digital inclusive finance index/100	The Peking University Digital Inclusive Finance Index (2011–2020)
Control variables	Economic development level	lnPGDP	Logarithm of GDP per capita	China City Statistical Yearbook
	urban infrastructure construction	INFRA	Per capita road mileage	China City Statistical Yearbook
	urbanization	URB	Urban population/ total population	China City Statistical Yearbook
	government financial support	lnGOV	Logarithm of local financial expenditures on scientific research	China City Statistical Yearbook
	industrialization	IND	Secondary industry’s added value/ GDP	China City Statistical Yearbook

This study employed panel data encompassing 30 Chinese provincial-level administrative regions (including provinces, autonomous regions, and municipalities directly under the central government) from 2011 to 2020. The period aligns with China’s 12th Five-Year Plan (2011–2015), which marked a strategic shift from “end-of-pipe treatment” to “whole-process control” in environmental governance, and the 13th Five-Year Plan (2016–2020), which reinforced green growth targets. Additionally, we terminated our sample in 2020 to avoid potential biases in the population mobility data that may have resulted from the COVID-19 pandemic in subsequent years. Tibet, Hong Kong, Macao, and Taiwan were excluded from the sample because of data availability considerations. The primary data mainly come from the *China City Statistical Yearbook*, *China Science and Technology Statistical Yearbook*, *China Industrial Statistical Yearbook,* and *China Financial Yearbook*. Digital inclusive finance data were derived from the Peking University Digital Inclusive Finance Index, compiled by the Digital Finance Research Center of Peking University in collaboration with the Ant Financial Services Group. In some cases, linear interpolation has been applied to handle the missing data. The detailed descriptive statistics for each variable are shown in Table 2.

**Table 2:** Descriptive Statistics of Variables.

Variable	Defination	Mean	SD	Min	Median	Max
lnGTFP	green total factor productivity	0.0300	0.0500	−0.0800	0.0100	0.260
POPM	population mobility	0.0300	0.890	−3.100	0	2.980
RD	Technological progress	0.120	0.110	−0.310	0.140	0.520
FIN	Financial development	0.0600	0.0800	−0.120	0.0400	0.500
DFIN	Digital inclusive finance	2.170	0.970	0.180	2.240	4.320
lnPGDP	Economic development level	10.84	0.440	9.710	10.79	12.01
INFRA	urban infrastructure construction	38.56	23.84	5.130	35.82	143.6
URB	urbanization	57.91	12.50	34.36	56.02	94.15
lnGOV	government financial support	4.300	1.040	1.320	4.180	7.060
IND	industrialization	2.870	0.620	1.710	2.780	4.660

## 4 Results

### 4.1 The spatial evolution of GTFP

#### 4.1.1 Whole network characteristics.

**Fig 1:**
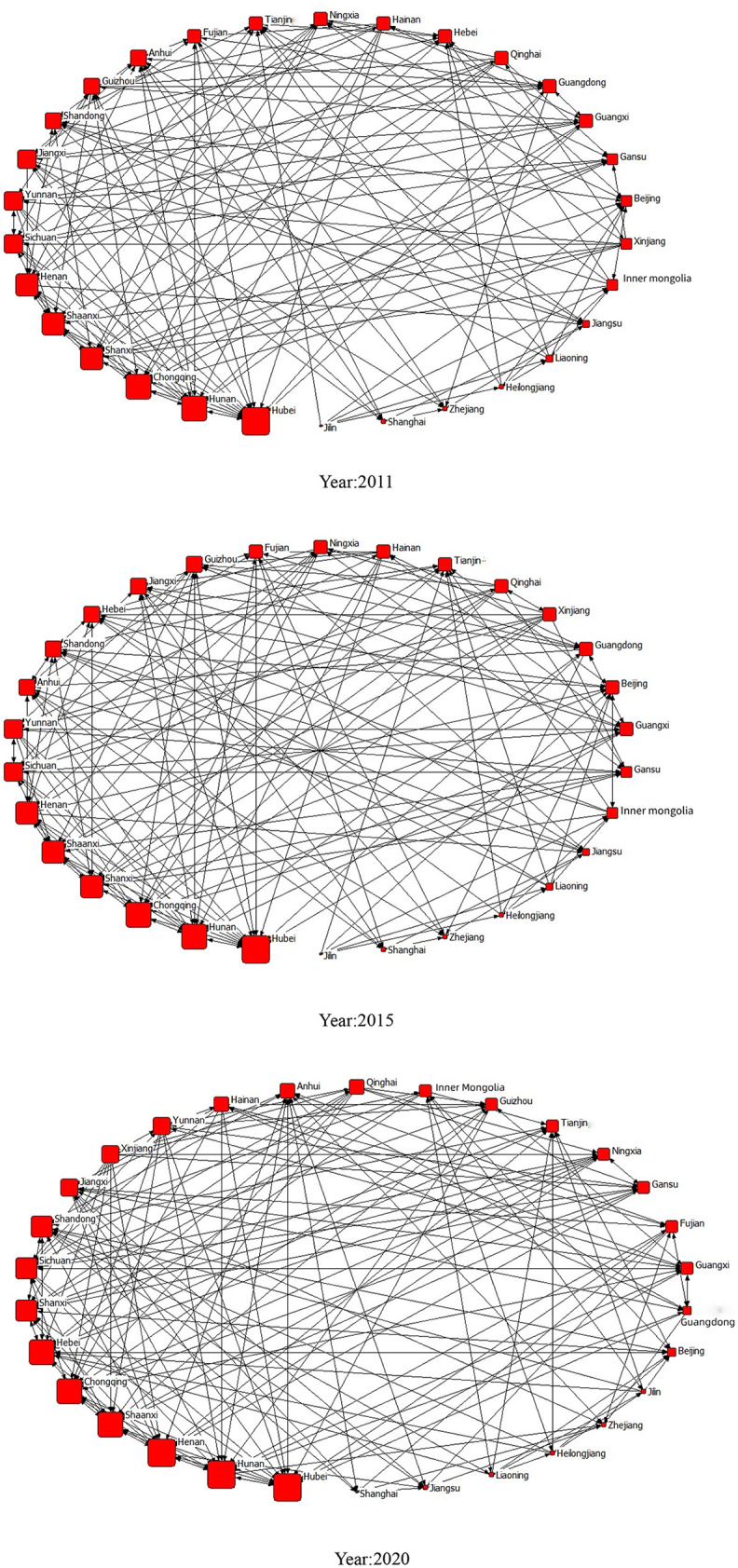
Social Network Diagram.

Using the UCINET visualization tool Netdraw, we plotted a spatial correlation network diagram of the point degree centrality of GTFP based on the constructed gravity matrix. Because of the stability of the spatial correlation network diagram, this study only reported the data for 2011, 2015, and 2020, as shown in Fig 1, where nodes represent provinces, node size reflects the level of point-degree centrality, and the links between nodes represent transportation paths for GTFP among provinces. The results suggest that all the provinces are connected within the GTFP development network, which displays a complex multithreaded spatial network structure. In 2011, the central provinces in the network were Hubei, Hunan, Chongqing, Shanxi, Shaanxi, Henan, and Sichuan, which maintained a dominant position in the social network structure of GTFP development. Provinces located in the eastern region, including Jilin, Shanghai, Zhejiang, Heilongjiang, Liaoning, Jiangsu, Inner Mongolia, and Beijing, exhibited smaller centrality in the spatial correlation network diagram. These provinces are characterized by loose connections with the network and are situated on the periphery. The positions of these provinces’ GTFP within the overall network remained relatively stable in 2015 and 2020.

**Fig 2:**
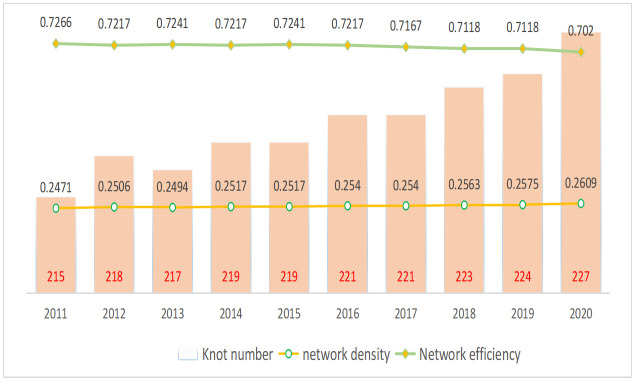
Network Density and Correlation Examination of GTFP.

Fig 2 illustrates the stability of the network density of GTFP from 2011 to 2020, which consistently hovers around 0.25. This value signifies a low level of interprovincial closeness in terms of GTFP. Concurrently, the number of network nodes continued to increase, indicating a robust national GTFP network structure with increased connectivity. These findings align with the expectations of sustainable development. Furthermore, the network efficiency of the GTFP has remained around 0.7, showing a decreasing trend over the years, which suggests that the stability of the interprovincial GTFP development network connections has been strengthening annually.

#### 4.1.2 Characteristic of ego-network.

Table 3 presents the degree, closeness, and betweenness centralities of each network node. The networked trend of GTFP is gradually becoming evident, with a national GTFP development pattern emerging in which the central and western regions support the development of the eastern regions. Specifically, western regions serve as the primary “source” of green resources flow, while eastern regions are the main “destination” for such flow. The central regions play a vital role as mediators in facilitating resource transfer.

**Table 3:** Local Network Centrality Analysis of GTFP.

	Point Centrality				Closeness Centrality		Betweenness Centrality	
	Indegree	Outdegree	Degree	Order	Degree	Order	Degree	Order
Hubei	13	7	14	1	61.702	3	53.176	8
Henan	12	10	14	2	65.909	1	133.026	2
Hunan	13	10	14	3	59.184	6	98.063	4
Shaanxi	11	9	13	4	63.043	2	64.602	7
Hebei	13	5	13	5	58.000	7	34.423	12
Chongqing	13	8	13	6	58.000	8	50.949	9
Sichuan	10	10	12	7	53.704	13	85.898	6
Shanxi	12	7	12	8	61.702	4	95.434	5
Shandong	10	9	12	9	56.863	9	152.301	1
Jiangxi	8	9	11	10	54.717	12	24.695	15
Yunnan	6	9	11	11	52.727	16	9.907	21
Xinjiang	1	11	11	12	56.863	10	8.876	22
Anhui	10	9	10	13	59.184	5	123.75	3
Hainan	3	10	10	14	49.153	21	7.242	23
Qinghai	2	10	10	15	52.727	15	34.822	11
Fujian	6	8	9	16	48.333	23	12.433	19
Gansu	7	6	9	17	51.786	17	4.518	27
Tianjin	9	4	9	18	50.877	18	4.851	26
Ningxia	6	7	9	19	54.717	11	22.67	16
Guangxi	7	9	9	20	48.333	22	30.582	13
Guizhou	7	7	9	21	50.000	20	10.953	20
Inner Mongolia	5	8	9	22	50.877	19	22.189	17
Beijing	8	5	8	23	47.541	26	27.153	14
Guangdong	5	8	8	24	47.541	25	17.436	18
Liaoning	4	7	7	25	43.939	30	40.263	10
Heilongjiang	3	7	7	26	43.939	28	0	29
Jiangsu	7	3	7	27	52.727	14	4.903	25
Jilin	3	7	7	28	43.939	29	0	30
Zhejiang	7	5	7	29	46.032	27	5.244	24
Shanghai	6	3	6	30	47.541	24	2.643	28
mean value	7.57	7.57	10	—	53.05	—	39.433	—

Specifically, there were 12 provinces with higher than average degree centrality, with 5 provinces each in the central and western regions. This observation indicates that the development of GTFP in these central and western provinces holds a more central position in the overall social network. These serve as the core network for GTFP improvement, which is consistent with the results shown in Fig 1. Furthermore, the provinces with a higher in-degree centrality are mainly scattered in eastern regions, representing the “destination” of resource flow within the network. In contrast, the western provinces have a higher out-degree centrality, acting as the “sources” of resource or information flow. These provinces possess strong radiative capabilities and can bring positive economic externalities to neighboring provinces. Notably, most provinces with an out-degree higher than an in-degree were located in the western regions, accounting for 46%. Provinces with a closeness centrality higher than the average included Henan, Anhui, Shanxi, Hubei, Hunan, Hebei, Shandong, Shaanxi, Chongqing, Sichuan, Jiangxi, Xinjiang, and Ningxia. This indicates that these provinces have a significant influence on the overall social network of GTFP development. They are the central actors in the social network and are less susceptible to the influence of other provinces, indicating good connectivity with other provinces in the network. Finally, 10 provinces exhibited higher betweenness centrality than average, with the central regions predominating. This indicates that the central regions have strong control over other provinces within the spatial correlation network of GTFP development and possess advantages in resources, funds, and technology.

### 4.2 Analysis of the total effect of population mobility on GTFP

#### 4.2.1 Pre-benchmark regression tests.

To address potential spurious regressions due to non-stationarity, this study applies the Levin-Lin-Chu panel unit root test. Table 4 shows that all variables reject the null hypothesis of a unit root, confirming the absence of nonstationarity in the data.

**Table 4:** Unit root test.

Variables	Adjusted t statistic	P-value
lnGTFP	−3.5099	0.0002
POPM	−13.1230	0.0000
lnPGDP	−1.5875	0.0562
INFRA	−67.0632	0.0000
URB	−5.0097	0.0000
lnGOV	−21.9883	0.0000
IND	−5.4455	0.0000

A co-integration test was conducted to examine the long-run equilibrium relationships among the variables. As shown in Table 5, the null hypothesis of no cointegration was rejected at the 5% significance level, confirming a stable long-run association.

**Table 5:** Kao cointegration test.

Kao test-statistics	Statistic	P-value
Modified Dickey-Fuller t	−1.9089	0.0281
Dickey-Fuller t	−2.3258	0.0100
Augmented Dickey-Fuller t	−2.6549	0.0040
Unadjusted modified Dickey-Fuller t	−2.0095	0.0222
Unadjusted Dickey-Fuller t	−2.3793	0.0087

To address the potential bias arising from cross-sectional dependence in the panel data model, we conducted cross-sectional correlation tests. As Table 6 shows, all three test specifications reject the null hypothesis of no cross-sectional dependence at the 1% significance level. Consequently, the subsequent empirical estimation must account for cross-sectional correlations.

**Table 6:** Cross-sectional dependence tests.

Test	Statistics
Pesaran CD test	8.081***
Pesaran scaled LM test	11.348***
Breusch-Pagan LM test	152.50***

Note: ***P < 0.01.

#### 4.2.2 Baseline regression.

Table 7 presents the results of the random effects model in column (1) and the regression analysis with national, destination region, and origin region fixed effects in columns (2) – (4). According to the Hausman test, the model with fixed effects performed better. The coefficient in column (2) is statistically significant and positive, indicating that population mobility has a positive impact on GTFP. The coefficients in columns (3) and (4) show that the effect is stronger in destination regions but not significant in origin regions, preliminarily verifying Hypothesis 1.

**Table 7:** Population Mobility and GTFP: Baseline Regression.

	(1)	(2)	(3)	(4)
	National regions	National regions	Destination regions	Origin regions
	lnGTFP	lnGTFP	lnGTFP	lnGTFP
POPM	0.0160^***^	0.0152^***^	0.0181^**^	0.0021
	(0.0036)	(0.0041)	(0.0079)	(0.0079)
lnPGDP	−0.0790^***^	−0.0931^***^	0.0040	−0.1408^***^
	(0.0192)	(0.0231)	(0.0366)	(0.0378)
INFRA	−0.0003	−0.0032^***^	−0.0033^***^	−0.0050^***^
	(0.0003)	(0.0007)	(0.0012)	(0.0011)
URB	0.5178^***^	0.2933^**^	0.3454^*^	0.9517^***^
	(0.0390)	(0.1437)	(0.1991)	(0.3239)
lnGOV	0.0007	−0.0032	−0.0205	0.0119
	(0.0064)	(0.0091)	(0.0131)	(0.0143)
IND	−10.3876^***^	−5.7952^**^	−6.8680^*^	−19.0456^***^
	(0.7861)	(2.8844)	(4.0016)	(6.5060)
_cons	0.7499^***^	0.8214^***^	−0.1065	1.3819^***^
	(0.1712)	(0.2307)	(0.3656)	(0.3539)
Year FE	NO	YES	YES	YES
Province FE	NO	YES	YES	YES
N	300	300	147	153
R-squared		0.6869	0.6756	0.7531

Notes: Standard errors in parentheses. *, **, and *** respectively represent that the variable coefficients have passed the significance test of 10%, 5%, and 1%, same as below.

In terms of the controlling variables, the coefficient of lnPGDP is significantly negative, indicating that the production model of sacrificing the environment for economic growth is no longer applicable. Additionally, the coefficient for INFRA is significantly negative, indicating that the current infrastructure development has not improved GTFP. One possible explanation for this observation is the high population density in destination regions, which has resulted in inadequate infrastructure. This limitation may reduce the potential benefits typically associated with population agglomeration. Similarly, insufficient infrastructure development in origin regions also fails to contribute to economic growth. Furthermore, the coefficient for URB is significantly positive, indicating that urbanization facilitates the transition from agriculture to non-agricultural sectors, thereby increasing labor productivity and the advancement of green technological innovation, which positively affects GTFP. However, the impact of lnGOV on GTFP remains inconclusive, because its coefficient does not exhibit a significant relationship with GTFP. Lastly, the coefficient of IND is significantly negative, suggesting that an economy dominated by heavy industries leads to environmental degradation with substantial pollutant emissions and negative environmental externalities.

#### 4.2.3 Discussion of endogeneity.

Two approaches were implemented to mitigate endogeneity issues caused by causal relationships. First, we expand the model into a dynamic panel using equation (3) and employ the generalized method of moments (GMM) for the regression analysis. In column (1) of Table 8, the GMM estimation with an AR (1) term was significant, whereas the AR (2) term was insignificant, which suggests the presence of a first-order serial correlation but not a second-order serial correlation in the model. Additionally, the Hansen test yielded a p-value greater than 0.1, rejecting the over-identification hypothesis of excessive instrument variables. Second, we employed a two-stage least squares (2SLS) approach in instrumental variable (IV) regression. In addition to including the lagged value of POPM (L.POPM) as an instrument, a measure of slope standard deviation in provincial capital cities (lnslope*year) was based on Zhang et al. (2021) [73]. This measure captures the geographical variation in the regional terrain, which is an important factor influencing population mobility. Moreover, slope is an objectively existing geographical condition shaped by a long-term history and does not affect GTFP. The 2SLS regression using L.POPM and lnslope*year as instruments is presented in columns (2) and (3) of Table 8. The first-stage results indicated that both L.POPM and lnslope*year had estimated coefficients that were significant at the 1% level and satisfied the relevance criterion. The F-statistic results suggest the absence of weak instrumental issues. The second-stage results revealed a significantly positive coefficient of population mobility. It is evident that population mobility has a positive impact on GTFP. Moreover, the coefficients estimated through GMM and 2SLS are larger than those obtained through ordinary least squares. This increase in the coefficient values may be attributed to the alleviation of endogeneity issues, leading to a more accurate estimation.

**Table 8:** Population Mobility and GTFP: GMM and 2SLS Estimates.

	(1)	(2)	(3)
	GMM	2SLS	2SLS
	lnGTFP	lnGTFP	lnGTFP
L.lnGTFP	0.2794^***^		
	(0.0859)		
POPM	0.0253^**^	0.0518^***^	0.0309^**^
	(0.0110)	(0.012)	(0.016)
lnPGDP	0.0189	−0.0601^**^	−0.0505^**^
	(0.0852)	(0.029)	(0.024)
INFRA	0.0003	−0.0025^***^	−0.0002
	(0.0021)	(0.001)	(0.000)
URB	0.3074^***^	0.9502^***^	0.5352^*^
	(0.0978)	(0.257)	(0.298)
lnGOV	0.0002	−0.0070	−0.0002
	(0.0396)	(0.011)	(0.004)
IND	−6.2008^***^	−19.0053^***^	−10.7679^*^
	(1.9694)	(5.164)	(6.010)
_cons	−0.1837	0.0320	0.5345^**^
	(0.7790)	(0.365)	(0.239)
Year FE	YES	YES	YES
Province FE	YES	YES	YES
N	270	270	300
R-squared		0.699	0.257

**Fig 3:**
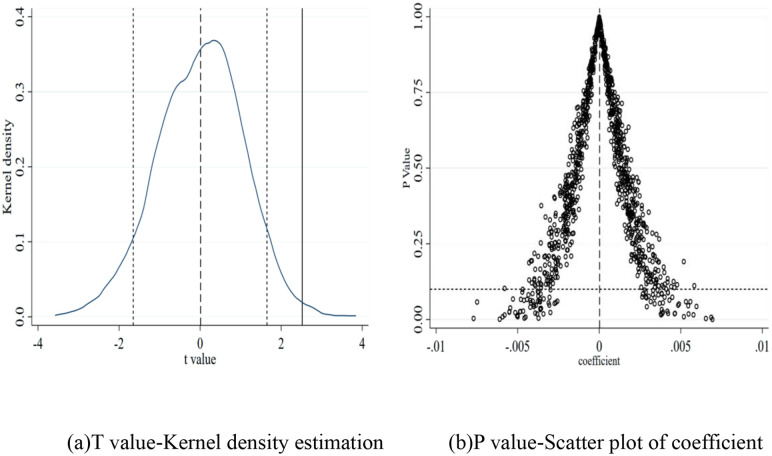
Population Mobility and GTFP: Placebo Test. (a)T value-Kernel density estimation (b)P value-Scatter plot of coefficient.

We conducted a placebo test to address endogeneity concerns. Specifically, the main explanatory variable, population mobility, was randomly sampled 1000 times to observe whether the kernel density plot of the coefficient or the observed values was distributed around zero and significantly deviated from the true value. The results are shown in Fig 3. Fig 3(a) indicates that the mean of the kernel density estimation of the t-values for the majority of POPM after randomization is concentrated around zero and deviates from its true value. In Fig 3(b), the horizontal dashed line represents P = 0.1, and most scatter plots are concentrated around zero and above the dashed line, indicating that most coefficients are insignificant at the 10% level or higher. Therefore, the promotion effect of population mobility on GTFP is not affected by other unobserved factors, and the results presented earlier are robust.

#### 4.2.4 Robustness test.

To further enhance the reliability of the conclusions, the following robustness tests were conducted: (1) Replacing the dependent variable. We replaced GTFP with Green efficiency (lnEFF) [74]. (2) The explanatory variable was replaced. As the main population involved in China’s population mobility is rural, the formula ([registered population – rural population]/ registered population) (MOB) was used to recalculate population mobility. (3) Outlier treatments. We trimmed the dataset by 1% to mitigate outlier effects. (4) Excluding policy interference. To account for potential policy shocks from China’s 2017 environmental policy reforms and their implementation lags, we restrict our analysis to the 2011–2018 subperiod. (5) Additional controls. We added two control variables to mitigate the omitted variable bias: fiscal decentralization (FISCAL), measured as the local-to-central government fiscal expenditure ratio [75]. Green finance (GFIN) is a composite index encompassing green credit, green investment, green insurance, green bonds, green support, green funds, and green equity [4]. As shown in columns (1) – (5) of Table 9, population mobility maintains a statistically significant positive relationship with GTFP, further confirming the robustness of the previous findings.

**Table 9:** Population Mobility and GTFP: Robustness Test.

	(1)	(2)	(3)	(4)	(5)
	lnEFF	lnGTFP	lnGTFP	lnGTFP	lnGTFP
POPM	0.0244^***^		0.0176^***^	0.0184^***^	0.0179^***^
	(0.0050)		(0.0039)	(0.0043)	(0.0039)
MOB		0.0040^***^			
		(0.0013)			
lnPGDP	−0.1131^***^	−0.1002^***^	−0.1046^***^	−0.1564^***^	−0.0980^***^
	(0.0301)	(0.0235)	(0.0227)	(0.0324)	(0.0233)
INFRA	−0.0044^***^	−0.0033^***^	−0.0035^***^	−0.0041^***^	−0.0028^***^
	(0.0009)	(0.0007)	(0.0007)	(0.0008)	(0.0007)
URB	0.5933^***^	0.3708^***^	0.5088^***^	0.7003^***^	0.3982^***^
	(0.0616)	(0.0446)	(0.0456)	(0.0679)	(0.0658)
lnGOV	−0.0089	−0.0125	−0.0060	−0.0111	−0.0073
	(0.0126)	(0.0100)	(0.0092)	(0.0107)	(0.0108)
IND	−11.7619^***^	−7.3489^***^	−10.1038^***^	−13.9631^***^	−7.8863^***^
	(1.2512)	(0.9015)	(0.9252)	(1.3751)	(1.3297)
FISCAL					−0.0086
					(0.1551)
GFIN					0.3415^**^
					(0.1613)
_cons	0.7708^***^	0.7008^***^	0.8859^***^	1.4973^***^	0.7207^***^
	(0.2797)	(0.2195)	(0.2105)	(0.3057)	(0.2124)
Year FE	YES	YES	YES	YES	YES
Province FE	YES	YES	YES	YES	YES
N	300	300	300	240	300
R-squared	0.6093	0.6095	0.6516	0.5997	0.6363

### 4.3 Heterogeneity analysis of population mobility on GTFP

Regional Perspective. Resource endowments and economic development vary among different provinces in China. Therefore, the country was divided into three regions: East, Central, and West, based on economic zones. The results in columns (1) – (3) of Table 10 indicate that population mobility significantly contributes to the improvement of the local GTFP in both the Eastern and Western regions. Moreover, the impact was stronger in the east, followed by the west, whereas the effect was insignificant in the Central region. This finding aligns with the current trend of population mobility in China, where the majority still move toward the Eastern region. The East boasts a more developed economy and higher resource endowments, which enable the greater realization of scale economies. Conversely, the Western region is gradually witnessing an increase in population mobility, whereas the Central region experiences an outflow of population.

**Table 10:** Heterogeneity Analysis: Regional Perspective.

	(1)	(2)	(3)
	Eastern regions	Central regions	Western regions
	lnGTFP	lnGTFP	lnGTFP
POPM	0.0290^***^	−0.0032	0.0197^***^
	(0.0068)	(0.0086)	(0.0054)
lnPGDP	−0.0792	−0.0003	−0.0292
	(0.0483)	(0.0437)	(0.0332)
INFRA	−0.0053^**^	−0.0057^***^	−0.0008
	(0.0026)	(0.0015)	(0.0008)
URB	0.5175^***^	0.6592^***^	0.4165^***^
	(0.0846)	(0.1562)	(0.1237)
lnGOV	−0.0080	−0.0138	−0.0156
	(0.0213)	(0.0150)	(0.0125)
IND	−10.2304^***^	−13.2604^***^	−8.3465^***^
	(1.7150)	(3.2046)	(2.5388)
_cons	0.3758	0.2314	0.3036
	(0.4898)	(0.3924)	(0.2745)
Year FE	YES	YES	YES
Province FE	YES	YES	YES
N	110	80	110
R-squared	0.6329	0.7772	0.6492

Industry stage perspective. Drawing on the theory of industrialization stages proposed by Konrad Zuse, we classified the development stages of regions based on the proportion of the output value of the tertiary industry to that of the secondary industry. If the ratio is less than 1, it indicates that the province is in the industrialization stage, whereas it suggests that the province has transitioned to the post-industrialization stage. The findings displayed in columns (1) and (2) of Table 11 demonstrate a significant positive relationship between population mobility during the post-industrialization stage and GTFP. This correlation can be attributed to the characteristics of traditional industries at the industrial stage, which are typically associated with labor-intensive operations, low productivity, and substantial pollution generation [48]. However, as industrial structures upgrade, population mobility toward tertiary industry leads to increased productivity, lower energy consumption, and reduced pollution, thereby contributing to the enhancement of GTFP.

**Table 11:** Heterogeneity Analysis: Stages Perspective.

	(1)	(2)
	Industrialization stage	Post-industrialization stage
	lnGTFP	lnGTFP
POPM	0.0109	0.0116^***^
	(0.0093)	(0.0044)
lnPGDP	−0.1544^**^	−0.0463
	(0.0611)	(0.0288)
INFRA	−0.0012	−0.0024^*^
	(0.0011)	(0.0013)
URB	0.4000^**^	0.3285^***^
	(0.1996)	(0.0543)
lnGOV	−0.0228	0.0148
	(0.0148)	(0.0144)
IND	−7.8618^*^	−6.5096^***^
	(4.0513)	(1.0968)
_cons	1.2839^**^	0.2187
	(0.6302)	(0.2646)
Year FE	YES	YES
Province FE	YES	YES
N	136	164
R-squared	0.3565	0.5134

### 4.4 Mechanism analysis of population mobility on GTFP

To investigate whether the effect of population mobility on GTFP is influenced by technological progress, financial development, and digital inclusive finance, a moderating effect model was employed. Columns (1) – (3), (4) – (6), and (7) – (9) of Table 12 display the moderating effect of technological progress, financial development, and digital inclusive finance on GTFP at the national level, as well as destination and origin regions, respectively. The coefficients of POPM×RD, POPM×FIN, and POPM×DFIN in columns (1), (4), and (7) indicate that, at the national level, technological progress has a significant positive moderating effect on the impact of population mobility on GTFP, whereas financial development and digital inclusive finance are insignificant, verifying Hypothesis 4. Notably, Table 12 reveals distinct regional patterns: In destination regions, both POPM×RD and POPM×FIN exhibit statistically significant positive moderating effects (columns 2, 5, and 8), whereas in origin regions, only POPM×DFIN demonstrates statistical significance (columns 3, 6, and 9), supporting Hypotheses 2 and 3.

**Table 12:** Mechanism: Technological Progress and Financial Development.

	(1)	(2)	(3)	(4)	(5)	(6)	(7)	(8)	(9)
	technological progress			financial development			Digital inclusive finance		
	National regions	Destination regions	Origin regions	National regions	Destination regions	Origin regions	National regions	Destination regions	Origin regions
POPM×RD	0.0451^***^	0.0693^*^	0.0097						
	(0.0143)	(0.0351)	(0.0283)						
RD	0.0321	0.0217	−0.0125						
	(0.0291)	(0.0364)	(0.0463)						
POPM×FIN				0.0383	0.1059^*^	−0.0529			
				(0.0392)	(0.0525)	(0.0366)			
FIN				−0.0048	−0.0090	−0.0650			
				(0.0251)	(0.0447)	(0.0499)			
POPM×DFIN							−0.0027	−0.0041	0.0280^***^
							(0.0033)	(0.0071)	(0.0075)
DFIN							−0.0186^**^	−0.0190	−0.0108
							(0.0091)	(0.0189)	(0.0099)
POPM	0.0148^***^	0.0127	0.0047	0.0164^***^	0.0178^**^	0.0074	0.0251^***^	0.0311^***^	−0.0781^***^
	(0.0033)	(0.0105)	(0.0065)	(0.0035)	(0.0075)	(0.0066)	(0.0079)	(0.0105)	(0.0272)
lnPGDP	−0.0903^**^	−0.0106	−0.1674^***^	−0.0887^*^	−0.0209	−0.1674^***^	−0.0662	0.0207	−0.1815^***^
	(0.0428)	(0.0417)	(0.0541)	(0.0443)	(0.0443)	(0.0524)	(0.0504)	(0.0585)	(0.0464)
INFRA	−0.0033^***^	−0.0033^*^	−0.0055^**^	−0.0033^***^	−0.0034^**^	−0.0054^**^	−0.0032^***^	−0.0036^**^	−0.0050^***^
	(0.0010)	(0.0016)	(0.0020)	(0.0010)	(0.0015)	(0.0020)	(0.0010)	(0.0017)	(0.0015)
URB	0.5306^***^	0.4514^***^	0.7465^***^	0.4862^***^	0.4328^***^	0.7208^***^	0.6322^***^	0.5655^***^	0.9971^***^
	(0.0878)	(0.0955)	(0.1726)	(0.0966)	(0.0840)	(0.1690)	(0.1086)	(0.1293)	(0.1842)
lnGOV	−0.0041	−0.0178	0.0095	−0.0067	−0.0206	0.0087	−0.0036	−0.0160	0.0130
	(0.0123)	(0.0134)	(0.0174)	(0.0120)	(0.0126)	(0.0178)	(0.0125)	(0.0137)	(0.0161)
IND	−10.5613^***^	−8.9951^***^	−14.9157^***^	−9.6583^***^	−8.6091^***^	−14.3927^***^	−12.5974^***^	−11.2937^***^	−19.9432^***^
	(1.7637)	(1.9199)	(3.5267)	(1.9423)	(1.6846)	(3.4456)	(2.1773)	(2.5963)	(3.7472)
_cons	0.7641^*^	0.0050	1.6671^***^	0.7360^*^	0.0994	1.6572^***^	0.5088	−0.2977	1.7262^***^
	(0.3989)	(0.4003)	(0.4640)	(0.4209)	(0.4208)	(0.4453)	(0.4696)	(0.5806)	(0.3449)
Year FE	YES	YES	YES	YES	YES	YES	YES	YES	YES
Province FE	YES	YES	YES	YES	YES	YES	YES	YES	YES
N	300	147	153	300	147	153	300	147	153
R-squared	0.6416	0.6460	0.6903	0.6331	0.6396	0.6948	0.6385	0.6404	0.7354

## 5 Discussion

China’s interprovincial GTFP exhibits growing social network connectivity with spatial agglomeration among neighboring provinces and uneven regional development. This pattern is reflected in the role of the middle and western regions in facilitating growth in the eastern region, which is likely attributable to the latter’s locational advantages and technological accumulation. Conversely, the relatively underdeveloped economies of the western provinces have prompted population migration toward prosperous coastal areas, facilitating the flow of green economic production factors. However, this dynamic also generates a siphoning effect, wherein regions with faster GTFP growth absorb production factors from other regions without generating a commensurate trickle-down effect [40]. Such asymmetrical factor flows underscore persistent regional disparities. The eastern region’s advanced infrastructure and favorable policy environment attract critical production factors, including talent, capital, and technology, which may enhance the GTFP in the short term but exacerbate long-term regional imbalances. The absence of a trickle-down effect suggests that GTFP growth in the east has not sufficiently translated into technology diffusion or investment returns in the central and western regions. This persistent disparity underscores the structural constraints in the interior provinces, where reliance on resource-based and labor-intensive industries limits their capacity for industrial upgrading and the absorption of technological spillovers. Moreover, China’s uneven GTFP development reflects broader deficiencies in the global governance system, such as the developmental divide between advanced and emerging economies and the inadequate provision of global public goods. Addressing these disparities necessitates policies that foster upward mobility for developing countries within the global value chain, particularly toward mid- and high-end sectors.

The fixed-effects model indicates that population mobility significantly promotes GTFP growth, consistent with the existing literature on productivity and environmental efficiency [19,22,23,43]. This positive relationship can be attributed to several mechanisms. Primarily, population mobility generates agglomeration economies, knowledge spillovers, and technological externalities. The resulting spatial concentration of economic activity enhances productivity by augmenting human capital, stimulating innovation, and improving labor market efficiency through superior resource allocation. These agglomeration dynamics furthermore generate network synergies that potentiate the diffusion of technology [8,40]. Furthermore, population mobility contributes to environmental sustainability by reducing operational and transaction costs and mitigating cross-border pollution [7]. However, these dynamics are not unique to China. For instance, mobility restrictions in Palestinian territories hinder job opportunities and economic exchange while increasing transportation-related energy consumption and emissions [20]. Notably, the GTFP benefits of population mobility are more pronounced in the destination regions. This asymmetric pattern aligns with classical economic development theory, where destination areas experience greater capital accumulation and economic growth through the absorption of surplus labor and productive factors from origin regions [41,42,45]. Globally, China exhibits a relatively low level of internal mobility, owing to its household registration system and other institutional constraints that have historically limited its intensity. Despite these limitations, a positive impact of green development was observed. This finding may also apply to other regions with widespread population mobility, such as Indonesia, Ethiopia, and Palestine. These regions are also experiencing urban-rural population shifts and rapid urbanization [20].

This study further uncovers the heterogeneous moderating effects of technological progress, financial development, and digital inclusive finance on the population mobility-GTFP relationship. Nationally, technological progress exhibited a significantly positive moderating effect, whereas financial capital was insignificant. This divergence stems from the role of population mobility in facilitating talent agglomeration and knowledge diffusion, which synergizes with technological progress [51,52]. Technology-intensive industries are particularly effective in upgrading production technologies, enhancing value chains, and facilitating manufacturing transformation [54]. In contrast, financial capital often flows disproportionately to polluting industries due to profit motives, potentially undermining GTFP gains [70], a pattern consistent with findings from BRICS economies, where financial development failed to improve environmental quality [76]. Notably, China’s current financial system faces certain challenges. Although the overall quantity of financial resources may not be lacking, the issue lies in the structure and efficiency of the system.

In addition, our sub-regional analysis revealed significant spatial heterogeneity in the moderating effects. While technological progress and financial development positively amplify the GTFP benefits of population mobility in destination regions, these moderating effects are statistically insignificant in the origin regions. This spatial asymmetry reflects fundamental disparities in regional development: origin regions typically suffer from constrained innovation capacity, limited financial resources, and inadequate infrastructure, creating the dual constraints of technological backwardness and financial scarcity. Consistent with the established literature, we find that technological and financial factors exert stronger positive influences on GTFP in developed economies [55]. Conversely, developing nations such as China and India experience productivity constraints owing to factor misallocation when their financial resources are limited [60]. China’s financial system presents particular challenges. While aggregate financial resources are abundant, structural inefficiencies persist. The system’s large-scale beliefs significant allocation inefficiencies, particularly in its bank-dominated architecture, which disproportionately favors state-owned enterprises. Digital inclusive finance has emerged as an effective moderator of the regions of origin. Population mobility facilitates regional digital economic development and enhances innovation capacity [9]. The rapid advancement of digital technologies has enabled structural adjustments in financial systems, allowing digital inclusive finance to optimize factor allocation and resource distribution. This mechanism is particularly valuable for promoting development in economically disadvantaged areas and offers important insights into achieving both common prosperity objectives and SDGs.

## 6 Conclusions and policy recommendations

With progressively aging populations and declining birth rates, the traditional model of relying on demographic scale for economic growth is yielding diminishing returns, compelling a shift in the fundamental factors of production underpinning sustainable development. While the conventional, extensive growth model—dependent on cheap labor and physical capital—must transition toward innovation- and efficiency-driven approaches, scholarly attention has predominantly focused on the roles of capital and technology in enhancing GTFP. In contrast, the potential of population mobility remains underexplored. As the most dynamic factor of production, mobility offers a novel activation mechanism with the inherent capacity to foster innovation, accumulate knowledge, and enhance structural adaptability. Compared to the protracted cycles of physical capital accumulation, the resource reallocation facilitated by population mobility offers a potentially more rapid and efficient pathway to optimization. Therefore, a central challenge for China and analogous economies is determining how to strategically harness population mobility during pivotal economic transformations to address shifting factor endowments, achieve efficient resource allocation, and ultimately unlock new drivers for sustainable growth in the coming era.

Given the crucial role of human factors in achieving coordinated economic, social, and environmental development, this study measures the GTFP in China using panel data from 30 provinces between 2011 and 2020. Subsequently, the social network characteristics of GTFP among the provinces were examined using the SNA method. Additionally, a fixed-effects model and a moderating-effects model were applied to empirically examine the impact of population mobility on GTFP and further explore the role of technological progress, financial development, and digital inclusive finance in enhancing this relationship. The results showed that China’s GTFP exhibited an agglomerative spatial pattern within its social network relationships, with green resources shifting from the central and western regions toward the eastern region. Population mobility boosts national GTFP. However, the effects were heterogeneous. Specifically, it was statistically significant in the destination regions, east and west, and in post-industrial-stage provinces. Furthermore, the moderating effect analysis reveals that technological progress strengthens the positive relationship between population mobility and GTFP, both nationally and in destination regions, whereas financial development amplifies the effect in destination regions. In contrast, digital inclusive finance positively moderates links in the origin regions.

Our empirical findings offer critical policy insights into global economic development and environmental governance. First, technology and information exchanges among provinces should be strengthened to foster a GTFP-oriented social network structure that enables better green resource integration and promotes sustainable development “demonstration effect”.

Second, regionally differentiated strategies for GTFP are essential. Green development policies should consider regional disparities in economic development and encourage knowledge transfer and technology diffusion from advanced to less-developed regions. For central and western regions with lower economic development levels and delayed green development, often characterized by population outflows, it is advisable to learn from sustainable development strategies from developed regions while leveraging local resources and optimizing workforce training to maximize the spillover effects.

Third, population mobility, accompanied by the flow of labor, capital, and technology, has become an endogenous driver of China’s economic growth. However, population mobility tends to lead to prosperity in destination regions, causing rural hollowing-out and widening the wealth gap, which is detrimental to achieving common prosperity. Therefore, regionally tailored population policies should be implemented with differentiated measures for the destination and origin regions. Destination regions should focus on reducing institutional barriers to population mobility and lowering the associated transaction costs. Conversely, origin regions require targeted incentives to attract return migration, while addressing human capital depletion through compensatory skill development initiatives.

Finally, region-specific policy innovations should be adopted to optimize the synergistic interplay between technological progress, financial capital, and population mobility. These measures should simultaneously enhance the efficiency of financial capital allocation and facilitate the optimal integration of production factors. This requires differentiated financial strategies tailored to regional characteristics: modernizing traditional financial systems in population destination regions and expanding digital inclusive finance platforms in origin regions.

Conventional growth models are increasingly threatening sustainability amid global resource depletion and environmental stress. GTFP offers a critical lens for assessing sustainable development, with population mobility emerging as a significant determinant. However, in practice, policymakers often face challenges in balancing economic, social, and ecological priorities when addressing population flows owing to complex socioeconomic structures and competing interests when formulating population mobility policies. This study examined the synergistic effects of production factors, including labor, capital, and technology, in advancing sustainable development, offering insights into the optimization of the tripartite equilibrium between economic growth, social welfare, and environmental preservation under resource constraints.

This study has several limitations that suggest directions for future research. First, constrained by data availability, our analysis utilizes aggregate population mobility statistics rather than disaggregated data on structural characteristics (e.g., age, gender, educational attainment, or migration distance). These demographic dimensions yield valuable insights into the heterogeneous green economic effects of population mobility. Future studies that incorporate micro-level data could substantially advance our understanding of these relationships. Second, our analysis does not fully account for the long-term dynamics of population mobility effects. As labor represents the most dynamic production factor, its impact on sustainable development likely follows non-linear trajectories over extended periods. Subsequent research employing longer time-series data could better capture these evolutionary patterns and temporal thresholds.

## Supporting information

S1 File Data of regression estimation. (XLS)

S2 File Data of placebo test. (XLS)

S3 File Code. (TXT)

S4 File Data of Fig 1, Fig 2 and Table 3. (XLS)

S1 Fig Fig 1. Social Network Diagram. Year 2011.
(TIF)

S2 Fig Fig 1. Social Network Diagram. Year 2015.
(TIF)

S3 Fig Fig 1. Social Network Diagram. Year 2020.
(TIF)

S4 Fig Network Density and Correlation Examination of GTFP. Fig 2. Network Density and Correlation Examination of GTFP.
(TIF)

S5 Fig Fig 3. Population Mobility and GTFP: Placebo Test. (a) T value-Kernel density estimation.
(TIF)

S6 Fig Fig 3. Population Mobility and GTFP: Placebo Test. (b) P value-Scatter plot of coefficient.
(TIF)
