# Supplementary figures and images for: Research on the impact of population mobility on green total factor productivity: A perspective from sustainable development
Source: PLoS One. 2026 Jan 2;21(1):e0337836. doi: 10.1371/journal.pone.0337836 (PMC12758790; doi:10.1371/journal.pone.0337836)

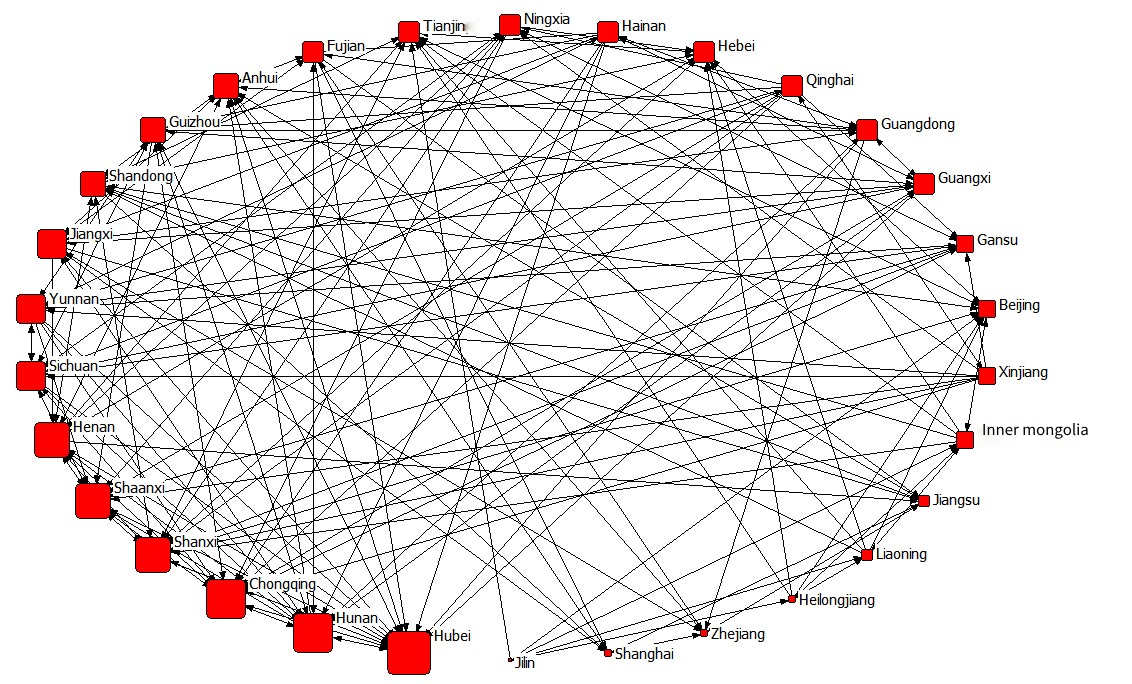

Supplement: S1 Fig — Year 2011. (TIF) [file pone.0337836.s005.tif]

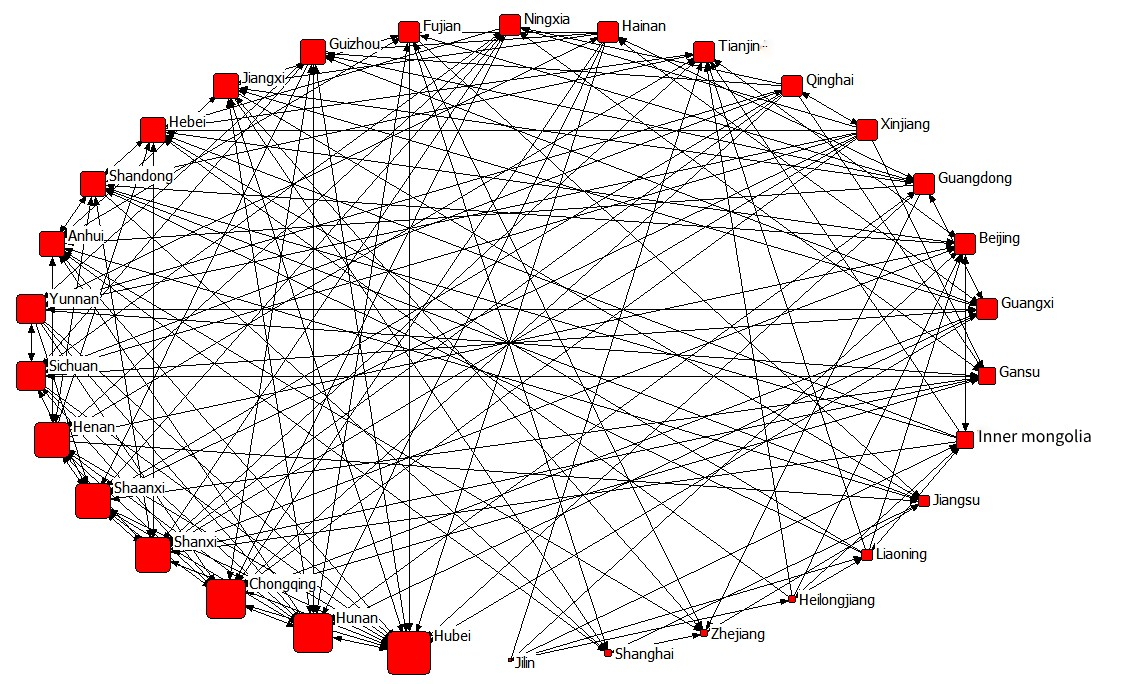

Supplement: S2 Fig — Year 2015. (TIF) [file pone.0337836.s006.tif]

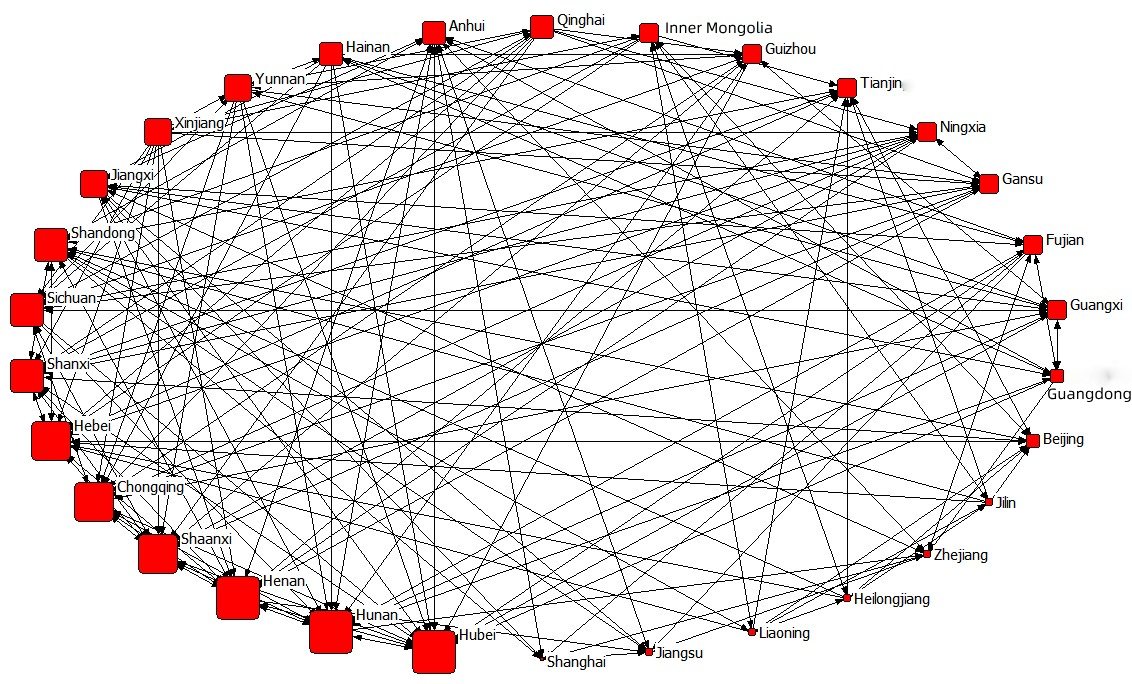

Supplement: S3 Fig — Year 2020. (TIF) [file pone.0337836.s007.tif]

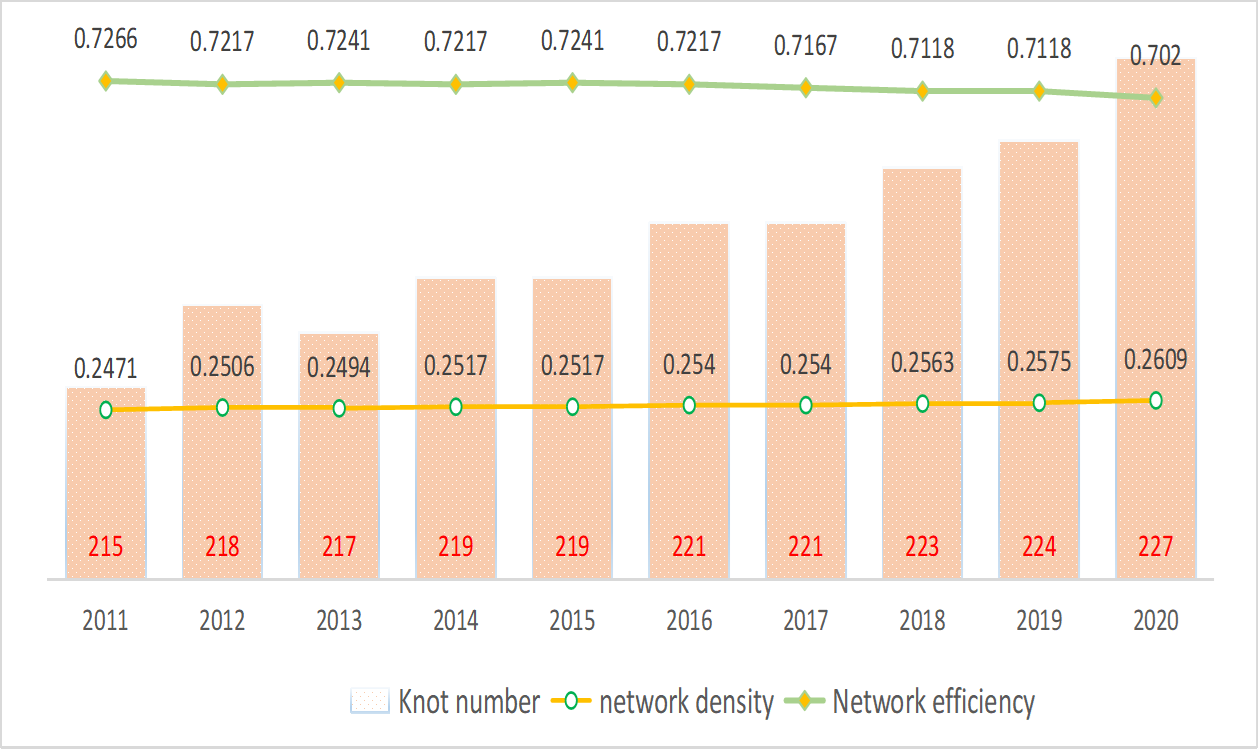

Supplement: S4 Fig — Fig 2. Network Density and Correlation Examination of GTFP. (TIF) [file pone.0337836.s008.tif]

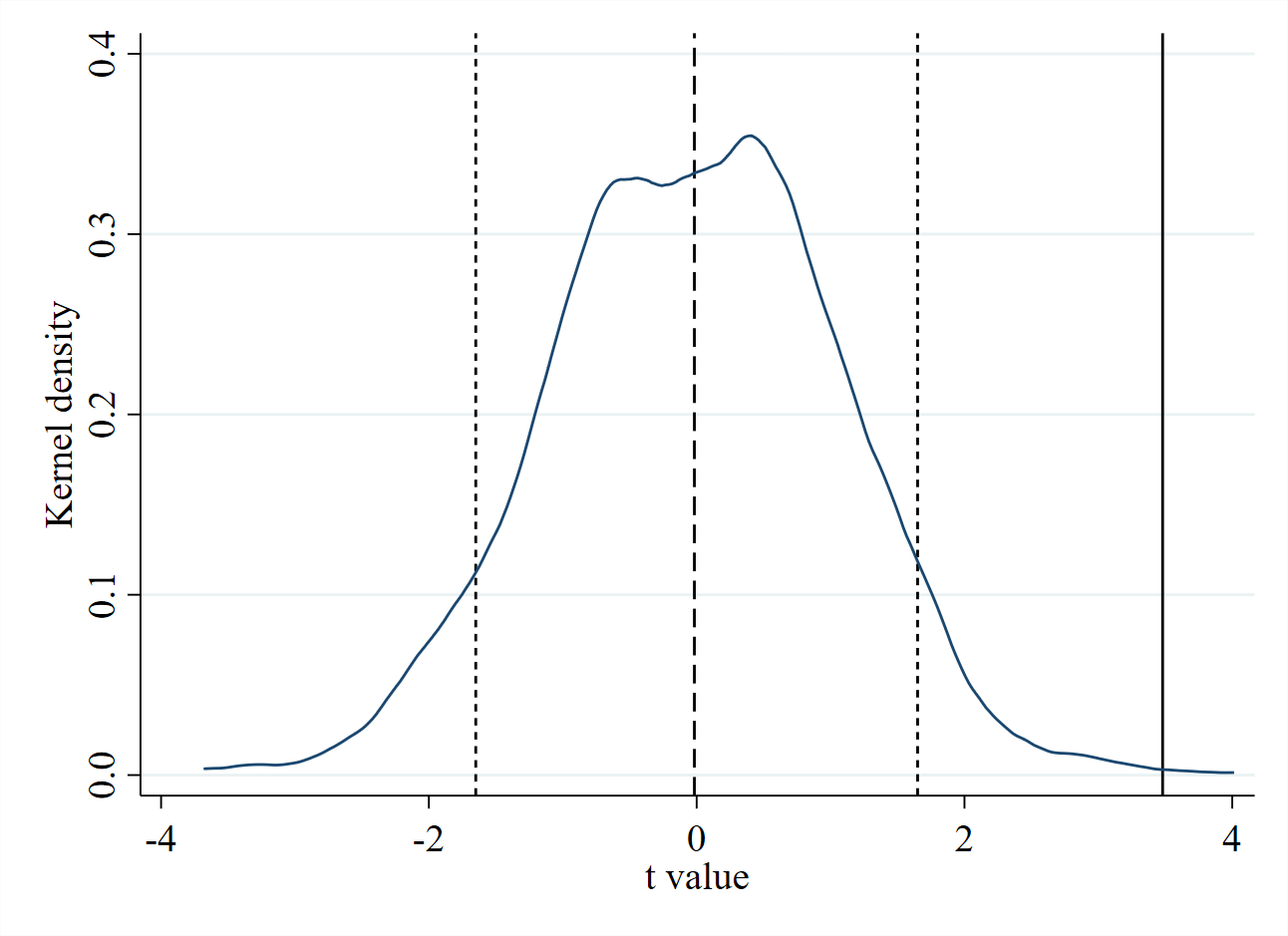

Supplement: S5 Fig — (a) T value-Kernel density estimation. (TIF) [file pone.0337836.s009.tif]

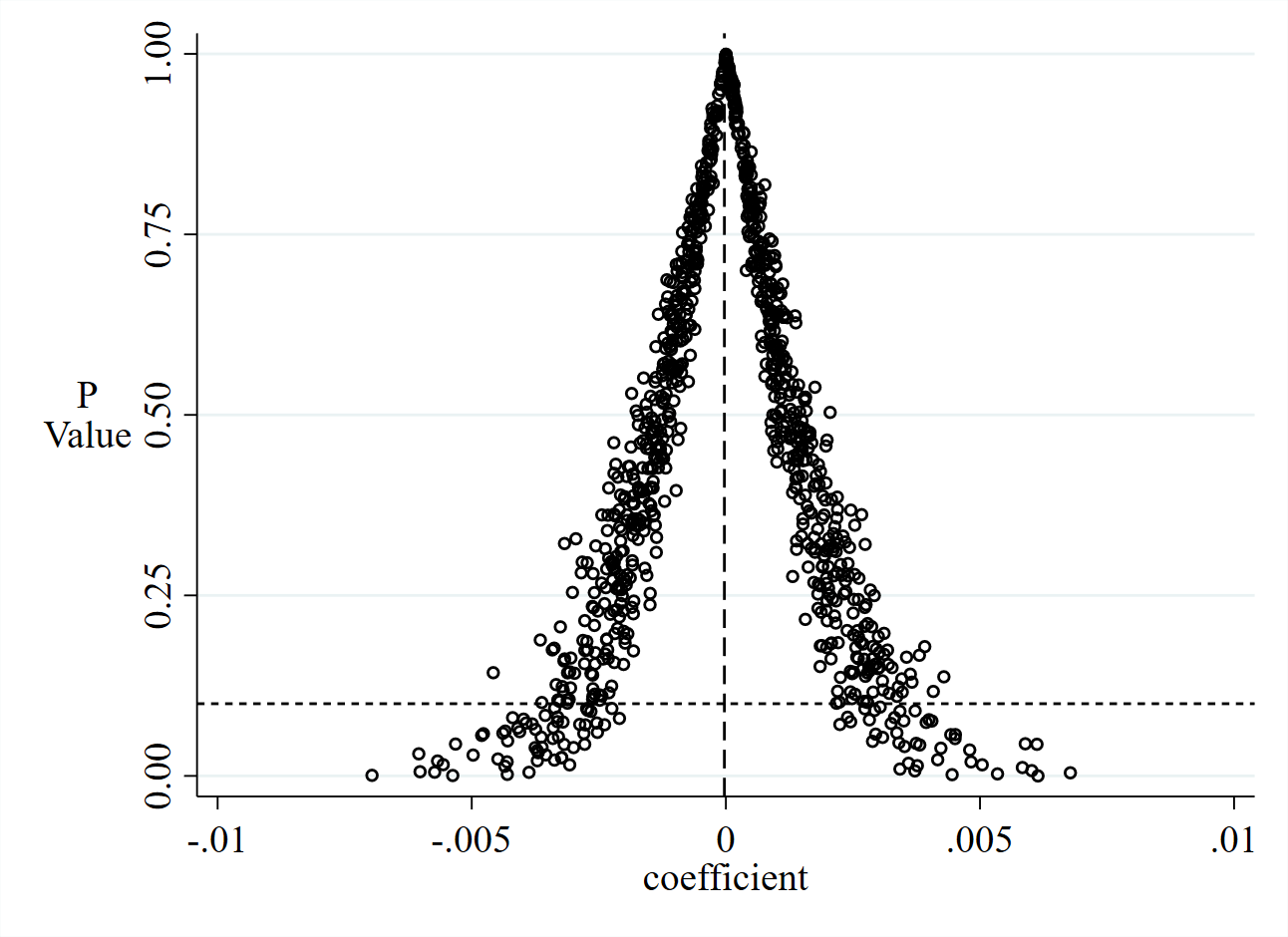

Supplement: S6 Fig — (b) P value-Scatter plot of coefficient. (TIF) [file pone.0337836.s010.tif]
